# Opportunities during COVID-19 towards achieving Universal Health Coverage

**DOI:** 10.7189/jogh.11.03115

**Published:** 2021-12-18

**Authors:** Keith Cloete, Mary-Ann Davies, Saadiq Kariem, Andrew Bouille, Krish Vallabhjee, Mickey Chopra

**Affiliations:** 1 Western Cape Government : Health, South Africa; 2 School of Public Health and Family Medicine, University of Cape Town, Western Cape South Africa; 3 World Bank, Washington, D.C., USA

Re-building a resilient health system that achieves universal coverage and financial protection is a daunting task [1]. Projections of public financing point to a significant squeeze on future health spending along with shifting political priorities suggest that postponing such a task until the COVID-19 pandemic has passed is not advisable [2]. Actually, many of the most durable and universal public health programs, such as the UK National Health System and New Deal in the US, were forged in the crucible of prolonged crisis. The ability of the state to draw together disparate partners and resources to offer universal and effective solutions can provide the political capital for fundamental reforms [3]. · Here we reflect on some early lessons on laying the foundations for a more universal and resilient health systems in the midst of fighting a pandemic.

The Western Cape province (WCP), South Africa covers an estimated population of 7 million and has been severely affected by the COVID-19 virus. By February 2021, around 270 000 positive COVID-19 cases had been reported and 15 000 deaths attributed to the virus [4]. The health system had to cope with two waves of outbreaks with a sharp rise during the second wave in December 2020 driven by a new and more infectious variant (**Figure 1**). The toll has been widespread not least amongst health workers with at least 8000 or 20% of the health workforce infected and more than 120 deaths [5]. This was exacerbated by global systemic challenges such as early shortage of diagnostics and effective treatments. Addressing this crisis required complex interventions for prevention, treatment of COVID and maintaining essential services – each of which provided opportunities to lay foundations for a universal and resilient health system.

**Figure 1:**
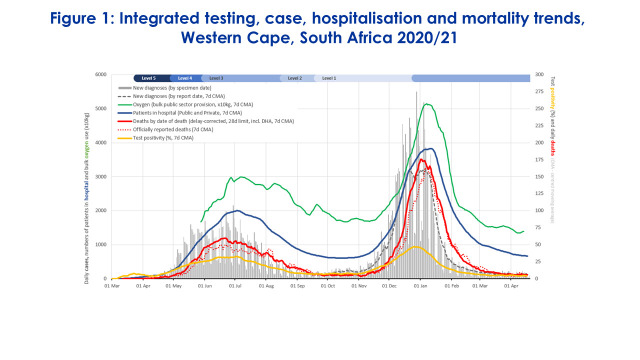
Integrated testing, case hospitalization and mortality changes, Western Cape, South Africa, 2020/21.

## SCALING UP DATA, CLINICAL AND PRIMARY CARE SYSTEMS

Establishing an authoritative and comprehensive data tracking system was an early imperative to both guide policy making and justify widespread preventive measures. The initial limits of testing capacity made early official estimates incomplete and allowed a multitude of complementary estimates, from research institutions, private providers and laboratories and global models, to proliferate. The WCP previously established a Provincial Health Data Centre (PHDC) that was consolidating person-level health data from the various clinical and clerical health information systems, including a web-based platform for clinical viewing of person-level data. The PHDC team, a mixture of data, public health and clinical experts, were able to integrate new data sets including from the private sector, linking and de-duplicating, implementing complex processing logic, and rapidly prototyping reports [6]. Alignment with cloud-based authentication enabled secure access from the public internet and then, within weeks of the first reported case, a public web based COVID-19 dashboard made daily estimates of new infections, deaths and their distribution by sub-district publicly available [7]. The sharing of this data helped to improve public confidence and trust with more than 2.15 million hits to date, and an average of 8000-10 000 hits per day during the both first and second wave peaks.

The second wave of the pandemic resulted in an unprecedent rise in the number of severely sick COVID-19 cases requiring hospitalization. This stretched the whole health care system requiring the establishment of field hospitals. The supply of medical oxygen becoming a particular challenge. Within a period of six weeks the oxygen requirements sharply increased from 12 to 82 tonnes of oxygen daily, beyond local production capacity. Increasing private sector production and distribution, ensuring that hospitals could safely manage increased oxygen deliveries and receive them in a timely manner required daily estimates of requirements and delivery plans based on clinical protocols, modelled estimates of new patients and existing hospital capacity triangulated with private production and delivery capacity which required the formation and functioning of a high-level team across public and private sectors. Key representatives from the clinical services, PHDC, private producer and logistics liaised daily to triangulate all the information and calibrate production, delivery of oxygen and clinical protocols across the different treatment facilities. Throughout this period there was no reported incident of hospital oxygen failure.

Several innovations were brought together to completely re-design the service delivery model required to free up health staff time and facility space to triage, manage and treat the sharp increase in COVID-19 cases. Adherence Clubs, that had started with providing community support for HIV patients [8], were expanded using community health workers and professional outreach workers (including telemedicine) to now support a majority of NCD patients to monitor their status outside of health facilities. The central pharmacy used previously imported Amazon warehouse technology and robotics to pre-package individual prescriptions [9]. Taxi and Uber drivers were then contracted to deliver directly to patients’ homes or community clubs. More than 1,6m medicine parcels have been delivered to date in the Cape Town metro region.

## FOUNDATIONS FOR UHC

Increased transparency and use of data, expanded clinical capacity, new models of care for chronic conditions will all be essential components of a more universal and resilient health system in the WCP. Making this possible during the crisis has required leveraging the existing state and civil society capabilities. Specific technical capabilities, ranging from data analysts and use of data through to skilled community workers, has been built on the back of consistent public health financing buttressed by ‘vertical’ disease program funding and technical support. This facilitated it playing a stewardship role in mobilizing and coordinating internal and external organisations and actors [10] (transcending sterile debates regarding public and private sector partnerships), balancing decentralized and centralized decision-making power, and establishing learning feedback loops to adjust the model to fit the environment [11]. Approaches such as New Public Management that shifted key functions to non-state actors has largely been rejected [12].

**Figure:**
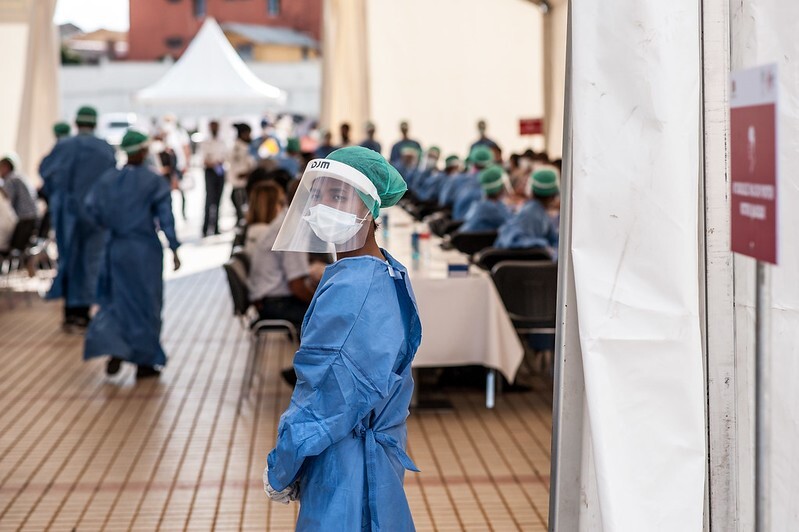
Photo: From the World Bank/Flickr collection.

Challenging the established institutional and governance arrangements was also required as traditional control and enforcing procedures did not facilitate the multi-partner collaboration and the adaptive governance required to respond to complex challenges [13]. Most acutely this required further recognition of the rights and safety of frontline workers including a widening definition and inclusion of this cadre.

Building state capabilities, changing expectations about accountability and legitimacy and, perhaps most challenging, changing the hierarchical culture of the state bureaucracies to allow more polycentric models of governance will be necessary to sustain the innovations and reforms [14].

Whilst necessary this is not sufficient. A more detailed exposition of the WCP experience would also reveal tensions and contradictions that were experienced. The development of the COVID-19 dashboard, for example, had to grapple with the need to encourage experimentation and decentralize decision making with the Province’s requirement of maintaining stability and standardization. The dashboard also clearly documented how the contours of apartheid still shaped the trajectory of the outbreak as it quickly spread and peaked in the poorer, largely black African and ‘Coloured’ neighbourhoods. Giving voice to those on the margins that is not merely tokenistic requires explicitly recognizing and challenging powerful political actors and the persistence of structural inequities [15]. Instead of an assumption of neutral relations as the starting point for collaboration, the differentiated impact of the COVID-19 virus suggests the need to acknowledge power imbalances and not just seek “work together” in an undifferentiated power field [16].

Ultimately the pandemic has, we believe, revealed a central, and yet neglected, tension regarding our understanding and operationalization of universality: can we achieve universality in the sense of universal population coverage and participation but in a manner that does not enforce a universality in the sense of adoption of a general point of view that leaves behind particular affiliations, feelings, commitments, and desires. The perpetual negotiation of the relation between those two senses of universality, whether read across differences of gender, socio-economic class, race or other social affiliation requires further exploration [17].

The philosopher, Walter Benjamin, writing during the turbulent Weimar Republic in Germany following the crisis of the WW1 and the subsequent 1918 Influenza Pandemic, spoke about the moment of awakening; the moment at which history emerges from the dream of a seamless continuity between past and present. The current pandemic and subsequent economic and social dislocation have provided us with our own ‘moment of awakening’ in which the hope of actualizing the future that will release the revolutionary potential of the present. The WCP COVID-19 response has significantly raised the political profile of public health in the province, enabling the Provincial Health department to play a stewardship role in guiding the whole of government response, and whole of society response. It has presented opportunities for leaders to engage with different groups and solicit their future aspirations and visions in an inclusive manner, to then identify early signs or potential of such a future in the present and to use the crisis to fast forward scale up the adoption of innovations and practices to form a bridge to a joint future.

## References

[1] Lal A Erondu NA Heymann DA Gitahi G Yates R Fragmented health systems in COVID-19: rectifying the misalignment between global health security and universal health coverage. Lancet. 2021;397:61-7. 10.1016/S0140-6736(20)32228-5 33275906 PMC7834479

[2] World Economic Outlook Update IMFJanuary 2021. Available: https://www.imf.org/en/Publications/WEO/Issues/2021/01/26/2021-world-economic-outlook-update. Accessed: 10 November 2021.

[3] Fukuyama F. The Pandemic and Political Order: It Takes a State. Foreign Aff. 2020. Available: https://www.foreignaffairs.com/articles/world/2020-06-09/pandemic-and-political-order. Accessed: 7 April 2021.

[4] Western Cape Government. Western Cape COVID-19 Dashboard. Available: https://coronavirus.westerncape.gov.za/covid-19-dashboard. Accessed: 7 April 2021.

[5] Moultrie T, Dorrington R, Laubscher R, Groenewald P, Bradshaw D. Correlation of Excess Natural Deaths with other Measures of the COVID-19 Pandemic in South Africa. Available: https://www.samrc.ac.za/sites/default/files/files/2021-03-03/CorrelationExcessDeaths.pdf Accessed: 7 April 2021.

[6] Boulle A Heekes A Tiffin N Smith M Mutemaringa T Zinyakatira N Data Centre Profile: The Provincial Health Data Centre of the Western Cape Province, South Africa. Int J Popul Data Sci. 2019;4:1143. 10.23889/ijpds.v4i2.1143 32935043 PMC7482518

[7] Mahomed H, Gilson L, Boulle A, Davies MA, Khan S, McCarthy K, et al. The evolution of the COVID-19 pandemic and health system responses in South Africa and the Western Cape Province – how decision-making was supported by data in District Health Barometer 2019/2020. In District Health Barometer. eds Massyn N, Day C, Ndlovu N et al. Durban: Health Systems Trust; 2020.

[8] Bango F Ashmore J Wilkinson L van Cutsem G Cleary S Adherence clubs for long term provision of antiretroviral therapy: cost effectiveness and access analysis from Khayelitsha, South Africa. Trop Med Int Healh. 2016;21:1115-23. 10.1111/tmi.12736 27300077

[9] Magadzire BP Marchal B Ward K Improving access to medicines through centralised dispensing in the public sector: a case study of the Chronic Dispensing Unit in the Western Cape Province, South Africa. BMC Health Serv Res. 2015;15:513. 10.1186/s12913-015-1164-x 26577831 PMC4650275

[10] Gilson L Barasa E Nxumalo N Cleary S Gouge J Molyneux S Everyday resilience in district health systems: emerging insights from the front lines in Kenya and South Africa. BMJ Glob Health. 2017;2:e000224. 10.1136/bmjgh-2016-000224 29081995 PMC5656138

[11] Janssen M van der Voort H Agile and adaptive governance in crisis response: Lessons from the COVID-19 pandemic. Int J Inf Manage. 2020;55:102180. 10.1016/j.ijinfomgt.2020.102180 32836637 PMC7309933

[12] Haque SM, Shyaka A, Mudacumura GM, editors. Democratizing Public Governance in Developing Nations: With Special Reference to Africa. London: Taylor & Francis; 2017.

[13] Rao NV Prashanth N Hebbar PB Beyond numbers, coverage and cost: adaptive governance for post-COVID-19 reforms in India. BMJ Glob Health. 2021;6:e004392. 10.1136/bmjgh-2020-004392 33558340 PMC7871228

[14] Wang C Medaglia R Zheng L Towards a typology of adaptive governance in the digital government context: The role of decision-making and accountability. Gov Inf Q. 2018;35:306-22. 10.1016/j.giq.2017.08.003

[15] Chopra M Inequalities in health in developing countries: Challenges for public health research. Crit Public Health. 2005;15:19-26. 10.1080/09581590500048218

[16] Funder M The Social Shaping of Participatory Spaces: Evidence from Community Development in Southern Thailand. J Dev Stud. 2010;46:1708-28. 10.1080/00220388.2010.492858

[17] Harvey D The Nature of Environment: The Dialectics of Social and Environmental Change. Social Regist. 1993;29:5621.

